# Optimization of calcium chloride and ultrasonication pre-treatment to mitigate the microbial load on fresh carrots using response surface methodology

**DOI:** 10.1016/j.ultsonch.2025.107311

**Published:** 2025-03-12

**Authors:** Muhammad Sameem Javed, Haq Nawaz, Fatima Filza, Muhammad Junaid Anwar, Faiz Ul Hassan Shah, Umair Ali, Muhammad Rizwan Tariq, Hammad Hafeez, Tawfiq Alsulami, Yash D. Jagdale, Robert Mugabi, Gulzar Ahmad Nayik

**Affiliations:** a Department of Food Safety and Quality Management, Faculty of Food Science and Nutrition, Bahauddin Zakariya University, Multan, Pakistan; b Department of Biochemistry, Bahauddin Zakariya University, Multan, Pakistan; c Department of Food Science and Technology, Faculty of Food Science and Nutrition, Bahauddin Zakariya University, Multan, Pakistan; d Department of Food Science and Technology, Faculty of Agriculture and Environment, The Islamia University of Bahawalpur, Bahawalpur, Pakistan; e Department of Food Sciences, Faculty of Agricultural Sciences, University of the Punjab-Lahore, Pakistan; f Department of Food Science & Nutrition, College of Food and Agricultural Sciences, King Saud University, Riyadh 11451, Saudi Arabia; g Department of Food Science and Nutrition, University of Minnesota, 1334 Eckles Avenue, St. Paul, MN 55108, United States of America; h Department of Food Technology and Nutrition, Makerere University, Kampala, Uganda; i Marwadi University Research Centre, Department of Microbiology, Marwadi University, Rajkot, Gujarat 360003, India

**Keywords:** Ultrasonication, Calcium chloride, Carrots, Response surface methodology, Total plate count

## Abstract

The quality and safety of fruits and vegetables are the major concerns of the food industry. The quality of these foods depends on their safety during storage and processing. The current research was designed to improve the quality by optimizing the safety parameters to minimize the microbial load of carrots during processing and storage. Three process variables including ultrasonication time, ultrasonication temperature, and calcium chloride concentration were optimized, each at five levels, to find the minimum optimal response of microbial load in terms of total plate count (TPC), total coliform count (TCC) and yeast mold count (YMC) using response surface central composite design. The selected factors showed statistically significant (p < 0.05) linear negative effect on TPC and linear positive effect on YMC. The total plate count, total coliform count, and yeast mold count were reduced to 6.117, 5.689, and 6.021 log CFU/mL respectively, under the optimum conditions i.e., ultrasonication time (17.97 min), temperature (43.885 °C), and CaCl_2_ concentration (181.211 mg/L). The non-significant interaction or quadratic effect of selected factors was observed on any of the studied responses. Concludingly, the reduction in studied parameters can improve the safety measurements of foods.

## 1 Introduction

The consumption of fruits and vegetables has seen notable increased across various regions, with specific numeric values reflecting these changes. For example the annual fruits and vegetables consumption was reached 200.1 kg per capita during the 2019–2021 period in Poland, marking a 5 % increase from earlier years [1]. In Mexico, between 1994 and 2014, there was a significant rise of 60 g (30.3 %) in fruits and vegetables consumption [2]. However, according to the Household Integrated Income and Consumption Survey (HIICS) 2015–2016, over half of the households in Pakistan do not meet the World Health Organization's recommended intake of 400 g per day per capita for fruits and vegetables [3]. These commodities' micronutrients, such as vitamins and minerals, are crucial to the human diet. They are popular worldwide for improving the growth and maintenance of the body [4]. Globally, the quality and safety parameters are centralizing the focus of researchers on modern storage, preservation, and processing techniques to ensure the availability of healthy and safe fruits and vegetables throughout the year. The quality parameters of vegetables can be considered regarding their nutritional value, microbial load and its influencing factors, and sensory and physicochemical properties [4,5]. Nutritionists prefer and recommend raw vegetables in a healthy diet plan [6]. Moreover, scientists played a major role by initiating the campaigns to create awareness among people and suggested consuming at least five servings of fruits and vegetables per day [7]. In Pakistan, the daily per capita intake of vegetables is about 134 g that is 66.5 % less than the recommendations (400 g/day) of GOP [8].

With the tremendous demand for fresh produce, people must have basic knowledge about the characteristics of fresh-cut produce [9]. The trimmed, peeled, or/freshly cut pieces of vegetables must be balanced and maintained by their freshness attributes and better quality properties of nutritional values after packaging [10]. Salads and fresh produce have become more attractive because of their nutritional benefits and are mostly consumed raw or after minimal processing. *Daucus carota* (carrot) is a main ingredient of salad along with other vegetables i.e., *Lactuca sativa* (Lettuce), *Cucumis sativus* (cucumber), *Lycopersicone esculentum* (tomato), *Spinacia oleracea* (spinach), *Brassica oleracea* (cabbage), *Raphanus sativum* (radish) [11]. Vending of cut salad vegetables, fruits, and sprouts is a common practice in Pakistan [12]. Outbreaks of human diseases due to the transmission of pathogenic microorganisms on vegetables usually result from fecal contamination which occurs due to the utilization of raw sewage or manure for fertilization, contaminated water for irrigation, and washing with contaminated ice, transportation and processing under unhygienic conditions [13]. Recent examples of outbreaks related to fresh produce include cases of *Escherichia coli* O157:H7 (spinach, lettuce), *Salmonella typhimurium* and *S. newport* (tomatoes, lettuce), *S. Thompson* (rocket) and hepatitis A (spring onion) [14]. Microflora is more often present on the upper surface of vegetables. However, only a few microorganisms can get entry inside the tissues of vegetables through obliteration of the surface and sometimes even in healthy flora [15]. Albeit, the risks linked with freshly produced vegetables cannot be mitigated completely, therefore risk limits are defined by various authorities [16].

Several decontamination approaches to decline the foodborne microbial count from the superficial area and to extend storage duration i.e., food handlers and industries use various kinds of washing and sanitizing techniques [17]. Simple water washing of raw commodities is the best technique to limit the microbial risk and residues on the surface of the commodity from the harvesting stage to the handling of fresh-cut processing [18]. In addition to washing techniques, refrigeration methods [19], suitable manufacturing techniques (e.g., the use of sharp cutting tools) [20] and an appropriately short time between processing and packing are important to improve and maintain the hygiene and quality of vegetable [21]. The results have demonastrated that calcium chloride alone helps to maintain firmness and reduce microbial growth by strengthening cell walls, but its effect on shelf life may be limited compared to combined treatments. Pretreatment of carrots with calcium chloride (CaCl_2_) solution facilitates the formation of calcium pectate, which enhances cell wall rigidity and reduces the respiration rate. Moreover, carrots lack endogenous calcium pectate-degrading enzymes [22]. Results have showed that ultrasonication alone can reduce microbial load and enzymatic activity but may not significantly impact texture or structural integrity without calcium reinforcement [23]. Furthermore, sonication effectively disrupts the DNA of surface microflora, thereby contributing to microbial inactivation and extending shelf life by synergistic effect. Ultrasonication works by producing high-frequency sound waves to create cavitation bubbles, which collapse and generate localized high temperatures and pressures, effectively disrupting microbial cell walls and significantly reducing total plate count (TPC) and yeast mold count (YMC) [24,25]. CaCl_2_ increase the antimicrobial activity, resulting the decrease in TPC and indicating its preservative efficacy [26]. A study suggested that the effect of ultrasound combined with slightly acidic electrolyzed water on removing *Listeria monocytogenes* biofilms from a glass surface, demonstrating enhanced biofilm clearance efficacy compared to individual treatments [27]. Moreover, the combined approach not only improves decontamination efficiency but also maintains the quality and safety of produce during storage [28]. Unlike chlorine treatments which can leave harmful residues, ultrasonication also minimizes chemical use [24]. Ultrasonication effectively targets a wider range of pathogens, specifically in the presence of organic matter, where chlorine treatment might be less effective [29]. The proposed method utilizing ultrasonication and CaCl_2_ for food processing depicts a flexible strategy that aligns with current industry practices. Large-scale ultrasonic baths or flow through highly designed tanks can efficiently treat bulk quantities of vegetables, allowing maximum control on processing parameters e.g., frequency and temperature [30]. The method is cost-effective, employing non-toxic, food-grade components like CaCl_2_, ensuring compliance with food safety regulations while maintaining affordability [31]. Ultrasonication effectively inactivates spoilage and pathogenic microorganisms, enhancing food safety [32]. Additionally, it improves the nutritional and sensory properties of food products, such as texture and flavor, making it suitable for various food categories [33]. However, while ultrasonication shows promise, challenges remain in optimizing parameters for different food matrices and ensuring consistent results across large-scale operations. Furthermore, Response Surface Methodology (RSM), is a type of statistical technique that evaluates and optimizes the process where there are various parameters, several reactions, and their responses are designated [34].

The objective of our study was the utilization of ultrasonication along with CaCl_2_ (sanitizing surfactant) to decrease microbial count from the surface of carrots. CaCl_2_ was combined with ultrasonication for decontamination and elimination of the probable microbial count. Optimization using RSM from the harvested carrots was done as these are contaminated by handling practices at harvesting along with microbial load in soil and mud through the utilization of ultrasonication along with CaCl_2_.

## 2 Materials and methods

### 2.1 Procurement of raw material

Raw fresh carrots were purchased from the vicinity of the local market of Multan City. The carrots were harvested in the first hours of the morning, saved in the ice buckets to keep the carrots fresh, and then transferred to the food processing laboratory of Institute of Food Science and Technology (IFSN), Bahauddin Zakariya University, Multan. The carrots were cleaned of dirt, dust, and other foreign particles by using brushes and scrubbers. After that carrots were filled in polyethylene, disinfected, and dried bags to avoid contamination and these bags were then stored in the refrigerators at 4 °C for further analysis. Moreover, all treatments and analyses were prepared and performed in the food microbiology and analysis labs of the institute under a controlled environment [35].

### 2.2 Response surface methodology (RSM) for experimental design of pretreatments and ultrasonication application

Three factorial central composite designs (CCD) of response surface methodology (RSM) were used to optimize the calcium chloride concentrations and ultrasonication treatments to mitigate or reduce the microbial load on carrots. RSM is a suitable technique to fit the quadratic surface that is considered to perform marvelously to streamline the process and consists of central factorial design. Three factors viz. X_1_:CaCl_2_ concentration (CCC; mg/L), X_2_:Ultrasonication time (USt; min), and X_3_:Ultrasonication temperature (UST; °C) were considered in this experimental design. The microbial count was determined by considering Yeast mold count (YMC; log CFU/mL), total coliform count (TCC; log CFU/mL), and total plate count (TPC; log CFU/mL). The input factors were contributing at five levels in permitted ranges. According to recorded literature, the current study was conducted to find the suitable range of each factor i.e. X_1_ was set at 0 mgL-^1^, 50 mgL-^1^, 100 mgL-^1^, 150 mgL-^1^ and 200 mgL-^1^, X_2_ was set from 0-20 min with the interval of 5 min and X_3_ was set 25–45 °C with an interval of 5 °C as per CCD design. The Microbial load response along with a set of factors (runs) designed by RSM depending on CCD is given in Table 1. By RSM design, 20 runs were considered and 6 replicating runs were designated as central points along with 14 axial points. The 2nd ordered equation of the polynomial quadratic model was utilized to evaluate the factors.

**Table 1:** Experimental values of microbial load in terms of TPC, TCC, and YM at various levels of input variables as suggested by experimental design.

Std.	Run	X_1_: CCC (mg/L)	X_2_ USt (min)	X_3_: UST (°C)	TPC (log CFU/mL)	TCC (log CFU/mL)	YM (log CFU/mL)
Control					8.69 ± 0.10	7.55 ± 0.48	7.40 ± 0.35
5	1	50	5	40	6.47	6.16	6.43
20	2	100	10	35	6.27	5.77	6.34
11	3	100	0	35	6.39	5.79	6.39
16	4	100	10	35	6.27	5.77	6.34
3	5	50	15	30	6.45	6.2	6.43
9	6	0	10	35	6.5	6.24	6.54
10	7	200	10	35	6.12	5.9	6.03
13	8	100	10	25	6.31	5.93	6.36
6	9	150	5	40	6.28	6.13	6.12
7	10	50	15	40	6.36	6	6.41
18	11	100	10	35	6.27	5.77	6.34
17	12	100	10	35	6.25	5.77	6.33
2	13	150	5	30	6.31	6.11	6.2
1	14	50	5	30	6.53	6.17	6.46
12	15	100	20	35	6.2	5.75	6.27
4	16	150	15	30	6.25	6.02	6.23
19	17	100	10	35	6.27	5.77	6.34
15	18	100	10	35	6.27	5.77	6.34
8	19	150	15	40	6.2	5.87	6.06
14	20	100	10	45	6.19	5.69	6.3
Mean ± SD					6.31 ± 0.05	5.93 ± 0.16	6.31 ± 0.03

CCC: Calcium Chloride Concentration, USt: Ultrasonication Time, UST: Ultrasonication Temperature, TPC: Total Plate Count, TCC: Total Coliform Count, YMC: Yeast Mold Count.

### 2.3 Ultrasonication and washings with CaCl_2_ concentration (CCC) treatments

The solutions were filled solitary in a sanitized and cleaned ultrasonication chamber (Elmasonic E 30H, 37 KHz) and carrots were placed inside the chamber, accordingly. The CCC solution was used in an ultrasonication chamber with adjusted time and temperature for treatment as per suggested runs were carried out. The method of Chen et al. was used in modified form for the placement of carrot samples and solution of calcium chloride in an ultrasonication chamber [36]. In short, the treatments were run for ultrasonication time from 0-20 min, at 37 KHz frequency and temperature from 25-45 °C with 40 ml/g water to raw material ratio by following suggested runs of CCD designed in RSM.

### 2.4 Microbial analysis of carrot samples

The random selection of carrot samples from the procured bags was carried out and microbiological analyses were performed to evaluate the microbial contamination levels before giving them any treatment. TPC, TCC, and YMC were the priority tests to detect the microbial load on carrot samples. Each experiment was conducted in triplicate by following the described method in FDA BAM [37].

### 2.5 Simultaneous study of ultrasonication and pretreatments

The ultrasonication of carrots was done by the pattern designed by RSM at a constant frequency (37 KHz) and alterations in temperature and time. Then washings of these ultra-sonicated carrots were done with different concentrations of CaCl_2_ formulated through RSM software.

### 2.6 Microbial analysis

#### 2.6.1 Media preparation and test tube setting

500 ml distilled water was taken in the flask and placed on a hot plate. Then, MacConkey agar and Plate count agar with quantities 26.55 g and 11.75 g were added in it with continuous stirring. Later on, flasks containing solutions and media were covered with aluminum foil and placed in an autoclave machine at 121 °C for 60 min to sterilize them [37]. Similarly, 9 ml PBS solution was added to the test tubes and also covered the test tubes with aluminum foil, and sterilized in an autoclave.

#### 2.6.2 Sample preparation for spreading

The surfaces of selected carrots were washed with PBS solution and then shifted in sterilized stomacher bags and the washed solution was completely homogenized in stomacher (Seward stomacher 400®UK) at 230 rpm for 5 min. Then 1 ml of this prepared sample solution was added to the PBS solution containing a test tube and the mixture was diluted up to 5 serial dilutions. After that, microbial analysis was done on this prepared inoculum [37].

#### 2.6.3 Total plate count (TPC)

The total plate count was measured by following the methods of AOAC [38]. In short, 20 ml plate count agar was poured into Petri dishes and placed petri plates in an air laminar flow chamber to solidify media under a sterile environment. Then, 100 µL inoculum was added to the plates having solidified media, and spreading was done with a glass spreader. Likewise, the plates of all treatments were prepared and incubation was done at 37 °C for 24 hrs. The colonies were counted in CFU/ml by placing the petri plates in the colony counter and then conversion was made in log base 10 to make the unit (log CFU/mL).

#### 2.6.4 Total coliforms count (TCC)

The prepared MacConkey agar media was taken in petri dishes. Then, placed it in a laminar airflow chamber and allowed it to solidify. Following the plate forming spread plate method, approximately 100 µL inoculum was added to the plates having solidified media. All remaining plates were arranged and prepared and incubation was done at 37 °C for 1 day (24 hrs) [37]. At the end, a Colony counter was used to count the colonies formed in petri plates.

#### 2.6.5 Yeast mold count (YMC)

Inoculation was done by following the described method of Kumar et al [35]. In this method, inoculation was done by spreading the inoculum on Petri plates using the glass spreaders [35]. The proper yeast and mold growth was obtained by following the instructions provided on the Petri plates. For instance, 1 ml solution was placed in an incubator at 28–30 °C for 24–48 hrs.

## 3 Statistical analysis

The statistical analysis of data was carried out by using a one-way analysis of variance as per the suggestion of RSM in the form of a polynomial quadratic equation. The response factors were predicted by creating the general polynomial regression equation to get the optimized results of actual input factors. The equation is given below

Y_i_ = β_o_ + β_1_X_1_ + β_2_.X_2_ + β_3_ X_3_ + β_11_X_1_^2^ + β_22_X_2_^2^ + β_33_X_3_^2^ + β_12_X_1_X_2_ + β_13_X_1_X_3_ + β_23_ X_2_X_3_.

In this equation Y denoted the predicted values generated by using the second-ordered polynomial quadratic design of response surface methodology, β_o_ represents the coefficient of regression that is persistent, β_1_, β_2_, and β_3_ depicts the coefficient of regression for the main linear effect of factor, β_11,_ β_22_, and β_33_ represented the quadratic effect of factors and β_12_, β_13,_ and β_23_ showed the interactional effects of the input factors. The current equation was utilized independently to calculate the predicted values in comparison to the values of an experiment for all responding variables to measure the precision, appropriateness, and consistency of the RSM presented a model to optimize the various variables of the process to obtain the optimized response levels. The calculations of Adjusted R^2^_,_ regression coefficient (R^2^), p-value probability (p < 0.05), lack of fitness (F-value) indicated the data points scattering, prominence and reliability of input factors/parameters, and lack of fitness in coefficient of polynomial quadratic regression, accordingly. The experimental acceptability was confirmed by the calculation of the coefficient of variance. The input factors and their responses were given in 3D graphical forms however the desirable numerical optimization was exhibited by counter graphs with the optimized values of the independent responses of variables. Design Expert 11.0 (Stat-Ease, Inc.), a statistical software was utilized to produce the experimental model, analyze the data and to optimize the procedures.

## 4 Results

The experimental values of initial microbial load in washings of fresh and processed carrots in terms of TPC, TCC, and YMC at various combinations of the selected processing factors as per experimental design are presented in Table 1. The TPC, TCC, and YMC of fresh carrots were found to be 8.69 ± 0.10, 7.55 ± 0.48, and 7.40 ± 0.35 log CFU/mL. The TPC, TCC, and YMC of processed carrots ranged from 6.12 to 6.53, 5.75 to 6.24, and 6.03 to 6.54 log CFU/mL with mean ± standard deviation of 8.69 ± 0.10, 7.55 ± 0.48, and 7.40 ± 0.35 log CFU/mL respectively. The one-way analysis of variance (ANOVA) of the experimental data using RSM showed a factor-dependent significant variation (*p < 0.05*) in TPC and YMC and non-significant variation in TCC at the selected combinations of treatment variables.

### 4.1 Response surface analysis and optimization of results

The effect of the selected processing factors on the initial microbial load of fresh carrots was optimized using quadratic polynomial response-surface models. The constructed model yielded the following polynomial regression equations to explain the relationship between the studied processing factors and the parameters of the initial load.

$$TPC\;\left(\frac{\log CFU}{mL}\right)=7.086-0.004X_{1}-0.013X_{2}-0.014X_{3}+0.025e^{-3}X_{1}X_{2}+0.035{e^{-3}X}_{1}X_{3}-0.250{e^{-3}X}_{2}X_{3}+7.091e^{-06}X_{1}^{2}+0.559e^{-3}X_{2}^{2}+0.109e^{-3}X_{3}^{2}$$

$$TCC\;\left(\frac{\log CFU}{mL}\right)=7.744-0.009X_{1}+0.050X_{2}-0.082X_{3}+0.100e^{-3}X_{1}X_{2}+0.040{e^{-3}X}_{1}X_{3}-0.001X_{2}X_{3}+0.038{e^{-3}X}_{1}^{2}+0.000X_{2}^{2}+0.001X_{3}^{2}$$

$$YMC\;\left(\frac{\log CFU}{mL}\right)=6.033+0.001X_{1}+0.012X_{2}+0.022X_{3}+1.00\;e^{-05}X_{1}X_{2}-0.100{e^{-3}X}_{1}X_{3}-0.040{e^{-3}X}_{2}X_{3}-6.363e^{-06}X_{1}^{2}-0.186e^{-3}X_{2}^{2}-0.186e^{-3}X_{3}^{2}$$

**Fig. 1:**
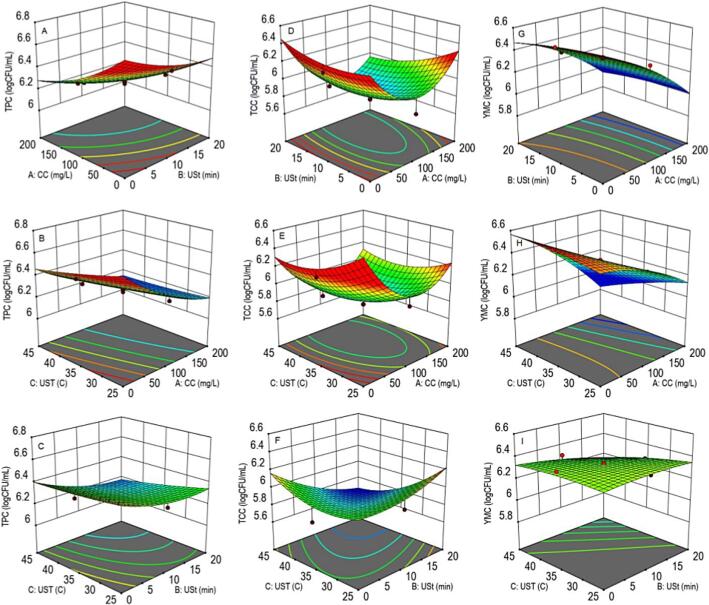
3-dimensional response-surface plots of microbial load carrots at the selected combinations of calcium chlorideconcentration, ultra-sonication time, and ultra-sonication temperature. TPC (A-C), TCC (D-F), and YMC (G-I). CC: Calcium chloride, USt, Ultra-sonication time, UST: Ultra-sonication temperature, TPC: Total plate count, TCC: Total coliforms count, YMC: Yeast mold count.

**Fig. 2:**
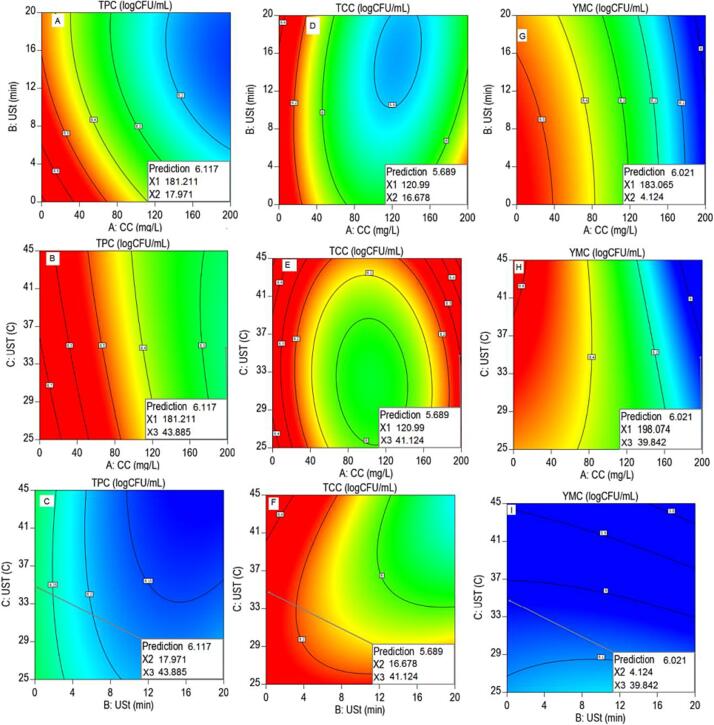
Optimum levels of input variables to attain the minimum optimal responses of TPC (A-C), TCC (D-F), and YMC (G-I). CC: Calcium chloride, USt, Ultra-sonication time, UST: Ultra-sonication temperature, TPC: Total plate count, TCC: Total coliforms count, YMC: Yeast mold count.

The above equations contained the estimates of coefficients to determine the main, linear, interaction, and quadratic effects of the input variables on the response parameters. The results of the analysis of variance in the experimental data are presented in Table 2. The measurement of lack of fit (F-value) and probability (p-value) values showed a significant positive main effect of the studied processing factors on TPC and YMC (F = 8.84, 38.63, and p = 0.001, <0.0001 respectively). Each of the selected factors showed a significant linear negative effect on TPC (F = 5.39–57.13, p=<0.0001–0.0426) and a positive linear effect on YMC (F = 7.04–315.01, and p=<0.0001–0.0241) while TCC remained unaffected after processing (F = 1.79, p = 0.188). However, none of the selected processing factors showed significant interaction or quadratic effects on any of the studied responses. The main, quadratic, and interaction effects of CaCl_2_ concentration CCC, USt, and UST on the TPC, TCC, and YMC were expressed in the 3-dimensional response surface plots (Fig. 1, Fig. 2 A-C, Fig. 1, Fig. 2 D-F, Fig. 1, Fig. 2 G-I respectively).

**Table 2:** Analysis of variance in TPC, TCC and YMC at selected combinations of the selected input variables.

**Source**	**TPC (log CFU/mL)**			**TCC (log CFU/mL)**			**YMC (log CFU/mL)**		
	**CE**	**F-value**	**p-value**	**CE**	**F-value**	**p-value**	**CE**	**F-value**	**p-value**
**Model**	7.087	8.84	0.0011	7.744	1.79	0.1880	6.034	38.63	< 0.0001
A-CCC (mg/L)	−0.005	57.13	< 0.0001	−0.009	3.01	0.1133	0.002	315.01	< 0.0001
B-USt (min)	−0.0138	12.30	0.0057	0.051	0.81	0.3893	0.013	7.04	0.0241
C-UST (°C)	−0.0145	5.39	0.0426	−0.082	1.74	0.2170	0.022	12.13	0.0059
AB	2.499e^-05^	0.12	0.7341	−0.00011	0.25	0.6279	10e^-06^	0.06	0.8193
AC	3.5e^-05^	0.24	0.6353	4e^-05^	0.03	0.8594	−0.0001	5.50	0.0409
BC	−0.00025	0.12	0.7341	−0.0018	0.67	0.4323	−0.0004	0.88	0.3702
A^2^	7.09e^-06^	3.09	0.1095	3.83e^-05^	9.51	0.0116	−6.36e^-06^	7.00	0.0245
B^2^	0.001	1.92	0.1962	0.0008	0.44	0.5201	−0.0002	0.60	0.4563
C^2^	0.0001	0.073	0.7925	0.0012	0.98	0.3460	−0.0002	0.60	0.4563
**R^2^**	0.8883			0.6174			0.9720		
**CV (%)**	0.8022			2.62			0.4775		
**AP (%)**	10.853			5.336			25.100		

CCC: Calcium chloride concentration, USt: Ultrasonication Time, UST: Ultrasonication Temperature, TPC: Total Plate Count, TCC: Total Coliform Count, YMC: Yeast Mold Count: R^2^: Regression coefficient, CV: Coefficient of variance, AP: adequate precision

The determination of the regression coefficient (R^2^ = 0.8883 and 0.9720) showed that almost 88.8 and 97 % of the variability in TPC and YMC could be explained through RSM, respectively. The relatively lower values of coefficient of variation (CV = 0.802, 0.4775 %) and higher values of adequate precision (AP = 10.853, 25.10 %) showed the reliability and adequacy of the employed response surface model.

**Fig. 3:**
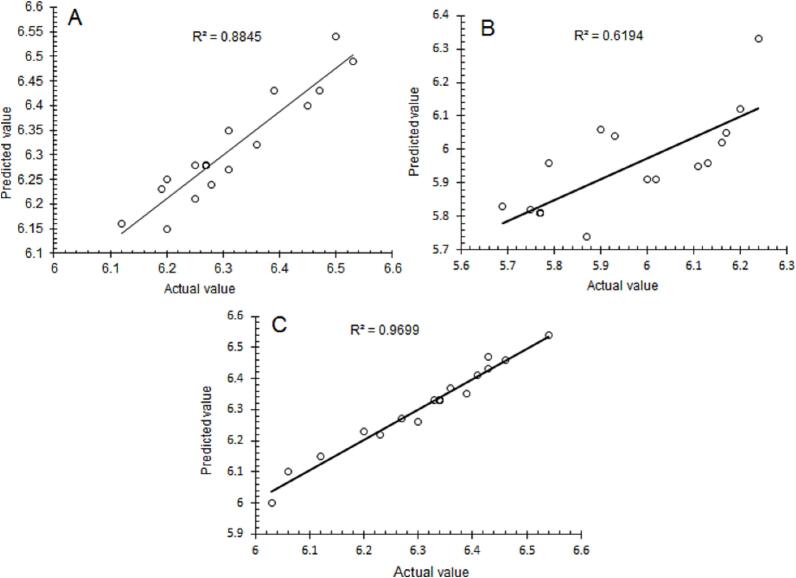
Correlation between actual and predicted values of the studied response (A) Total plate count (B) Total coliforms count, and (C) Yeast mold count.

The polynomial regression equations were used to calculate the predicted values of TPC, TCC, and YMC at various combinations of CCC, USt, and UST. The predicted values of the responses were plotted against the actual ones to determine the applicability of the employed response-surface model. The plot showed a strong correlation between the actual and predicted values of the TPC and YMC with relatively higher values of correlation coefficient (R^2^ = 0.8845 and 0.972 respectively) (Fig. 3a–c). The relatively higher values of R^2^ suggest that the constructed model can be successfully applied to study the relationship between the processing factors including CCC, USt, and UST, and the initial microbial load in terms of TPC and YMC.

The numerical optimization of the selected factors showed an optimum reduction in plate count with minimum levels of TPC (6.117 logs CFU/mL) at CCC: 181.211 mg/L, USt: 17.97 min, and UST: 43.885 °C. The minimum level of TCC (5.689 log CFU/mL) was observed at CCC: 120.99 mg/L, USt: 16.678 min, and UST: 41.124 °C while that of YMC (6.021 log CFU/mL) was found at CCC: 183.065 mg/L, USt: 4.124 min, and UST: 39.842 °C.

## 5 Discussion

The microbial deterioration of the vegetables during storage is among the major issues that increase the pathogenic load on the outer and inner portions of the vegetables. A study depicted the impact of a personalized microbial association on the efficiency of aerobic digestion of food waste [39]. Carrots depicted a diverse range of bacteria, e.g., *Pseudomonas, Enterobacter, and Lactobacillus,* which are present on their surfaces [40,41]. Studies indicate that carrots grown in manure-fertilized soil showed a significant overlap in antibiotic resistance genes (ARGs) with their surrounding soil, suggesting a unique transfer pathway of microbes from soil to carrot [42]. The microbs on carrots can change dramatically under different storage conditions. In contrast, non-ventilated storage conditions lead to a predominance of *Lactobacillales* and *Enterobacteriales,* which are associated with spoilage [41]. Unlike carrots, other vegetables may not exhibit the same level of ARG transfer from soil, nor do they typically harbor the same bacterial diversity. For example, the endophyte composition in carrots is influenced by both genotype and management practices, which may not be as pronounced in other crops [43]. This pathogenic load can be a leading cause of various chronic diseases. Several strategies are being utilized to preserve the nutritional value of fruits and vegetables during storage and further processing. In continuity with the past studies, the present research showed the effects of ultrasonication treatment in combination with the sanitizing surfactant (CaCl_2_) to reduce pathogenic loads such as TPC, TCC, and YMC. A study suggested that ultrasonic treatment promotes oxidation, indicated by decreased sulfhydryl and increased carbonyl groups, enhancing gel network stability [44].

In the present study, the significant linear negative effect of CCC on TPC suggested that TPC is decreased with an increase in CCC which may be attributed to the antimicrobial effect of calcium chloride as a preservative [45,46]. The results indicated that the utilization of decontamination process non-significantly reduce the micronutrient content of the carrots. Specifically, the levels of essential vitamins i.e., vitamin C and minerals auch as potassium were significantly preserved after the treatment. However, there was a slight reduction in certain bioactive compounds, which can be attributed to the physical nature of ultrasonication. Furthermore, the decontamination process also reduced microbial load without compromising the valuable micronutrient content of carrots to significant extent. The quadratic negative effect of CaCl_2_ concentration (CCC) on YMC suggests that calcium chloride shows positive effects against YMC at relatively higher concentrations. The USt and UST also showed a significant linear decrease in TPC suggesting that the ultrasonication of the samples at a relatively higher time (15–20 min) and temperature (40–45 °C) is significantly effective against microbial growth. This may be due to the higher efficiency of ultrasonication (US) treatment to destroy the microbes and reduction in TPC, molds, and yields [47]. It has been reported that ultrasonication of the sample results in a decrease in microbial load due to the formation of free radicals and localized temperature [48]. These results are in agreement with those reported earlier [49]. Significant decreases in total plate counts, yeast, and molds of apple juice treated with US alone have been reported [50].

The linear positive effect of CCC on YMC indicates an increase in YMC in response to an increase in CCC suggesting a poor effect of calcium chloride against yeast mold. The CCC-dependent increase in YMC may also be attributed to the calcium chloride-induced increase in moisture on the surface of carrots that can lead to an increase in YMC [51]. The USt and UST also showed a significant linear positive effect on YMC. It has been reported that ultrasonication of the sample results in a decrease in microbial load/(TPC) due to the cavitation that occurs by chemical reactions and physical effects on the commodity [52]. Cavitation generates high shear forces that can disrupt cell membranes, leading to cell lysis i.e., *Bacillus cereus* and *Oscillatoria brevis* were effectively destroyed within seconds of sonication due to mechanical disruption [53]. Gram-negative bacteria, such as *Pseudomonas aeruginosa,* exhibit higher susceptibility to ultrasonic treatment compared to Gram-positive bacteria like *Staphylococcus aureus*. The protective capsule of Gram-positive bacteria can shield them from cavitation-induced damage [54]. Different studies depicted that Gram-negative species often experienced greater reductions in viability, the effectiveness varied among different strains, indicating that structural differences also play a role in their susceptibility to cavitation [55].

The observed linear positive effect of USt and UST on YMC may be due to cavitation [47]. However, the non-significant main, linear, interaction, and quadratic effects of the studied factors on TCC also suggest that the selected processing factors are not effective in reducing the coliform count of the vegetables, particularly carrots, which might be due to multiple reasons such as coliforms can develop biofilms that protect them from disinfectants and can thrive in aquatic environments, complicating treatment efforts [56]. The exposure times used in CCC, USt, and UST may not be adequate for effective disinfection. The studies suggested that the increase in chlorine concentrations (up to 10 mg/L) and longer contact times (up to 30 min) have shown the improved efficacy in coliforms reduction [57].

The present results suggest that the use of the selected processing factors including CCC, USt, and UST are effective in reducing the microbial plate count of the samples but ineffective against coliform and yeast molds. The results of response-surface optimization suggest that the developed response-surface model can be successfully applied to study the relationship between the processing factors including CCC, USt, and UST, and the initial microbial load in terms of TPC and YMC. The correlation between the actual and predicted values of the response with relatively higher values of R^2^ and AP and lower values of CV also advocate the adequacy, reliability, and applicability of the constructed model.

The current technique might have some limitations e.g., extended processing time periods and high equipment costs during utilizing it on large scales decontamination processes. High energy and water usage and disposal concerns increase operational costs and environmental impact. Ensuring uniform treatment and maintaining product quality becomes difficult at scale, while equipment scalability and regulatory approvals add to the complexity. These limitations highlight the need for further optimization to make the process efficient and commercially viable.

## 6 Conclusion

The present study demonstrated that CCC and ultrasonication (USt) treatments effectively reduced the microbial load on carrots. A linear decrease in total plate count and yeast and mold count was observed with the increase in levels of CCC and USt. The optimized treatment conditions, identified in this study, can be applied to carrots and potentially other vegetables having similar surface characteristics to improve microbial safety during storage. However, further studies are needed to confirm their effectiveness on different vegetables. These findings contribute to the advancement of post-harvest management strategies, supporting the maintenance of freshness, quality, and extended storage life of vegetables.

## CRediT authorship contribution statement

**Muhammad Sameem Javed:** Writing – review & editing, Writing – original draft, Software, Formal analysis, Data curation, Conceptualization. **Haq Nawaz:** Writing – review & editing, Writing – original draft, Methodology, Investigation, Data curation, Conceptualization. **Fatima Filza:** Writing – review & editing, Methodology, Investigation, Formal analysis, Data curation. **Muhammad Junaid Anwar:** Writing – review & editing, Software, Project administration, Methodology, Investigation, Conceptualization. **Faiz Ul Hassan Shah:** Writing – review & editing, Visualization, Resources, Methodology, Investigation, Formal analysis. **Umair Ali:** Writing – review & editing, Visualization, Validation, Resources, Methodology, Formal analysis, Data curation. **Muhammad Rizwan Tariq:** Writing – review & editing, Validation, Supervision, Software, Resources, Project administration, Data curation, Conceptualization. **Hammad Hafeez:** Writing – review & editing, Resources, Methodology, Investigation, Formal analysis, Data curation. **Tawfiq Alsulami:** Writing – review & editing, Writing – original draft, Validation, Investigation, Funding acquisition, Conceptualization. **Yash D. Jagdale:** Writing – review & editing, Validation, Resources, Investigation, Formal analysis. **Robert Mugabi:** Writing – review & editing, Validation, Project administration, Funding acquisition, Formal analysis. **Gulzar Ahmad Nayik:** Writing – review & editing, Visualization, Validation, Supervision, Software, Resources, Investigation, Formal analysis.

## Declaration of competing interest

The authors declare that they have no known competing financial interests or personal relationships that could have appeared to influence the work reported in this paper.
